# Testosterone differentially modulates the display of agonistic behavior and dominance over opponents before and after adolescence in male Syrian hamsters

**DOI:** 10.3389/fnbeh.2025.1603862

**Published:** 2025-07-10

**Authors:** Arthur J. Castaneda, Conner J. Whitten, Tami A. Menard, Cheryl L. Sisk, Matthew A. Cooper, Kalynn M. Schulz

**Affiliations:** ^1^Department of Psychology, University of Tennessee Knoxville, Knoxville, TN, United States; ^2^Department of Psychology, Michigan State University, East Lansing, MI, United States

**Keywords:** testosterone, puberty, adolescence, agonistic, aggressive, submissive, dominance, social behavior

## Abstract

The current study investigated the influence of testosterone on agonistic behavior and dominance over an opponent before and after adolescence in male Syrian hamsters (*Mesocricetus auratus)*, and tested the hypothesis that shifts in behavioral responsiveness to testosterone occur across adolescent development. We predicted that testosterone-dependent modulation of attacks decreases following puberty, and that flank marking behavior in response to testosterone increases following puberty. Prepubertal (14 days of age) and adult subjects (52–62 days of age) were gonadectomized and immediately implanted with testosterone propionate (TP) or vehicle pellets. Fourteen days later, agonistic behaviors were assessed in a neutral arena with age-matched testosterone-treated opponents. TP treatment increased attacks and dominance over an opponent in prepubertal but not adult males, supporting the hypothesis that testosterone-dependent modulation of aggression decreases following puberty. TP increased flank marking behavior in adults, but failed to increase flank marking in prepubertal subjects, supporting the hypothesized increase in testosterone-dependent modulation of flank marking after puberty. Thus, we provide here evidence that changes in agonistic responses to steroid hormones occur across puberty and adolescence in male rodents, much like the well-established shifts in neuroendocrine and reproductive behavioral responses to steroid hormones that occur pre- to post-pubertally. These findings may have implications for early pubertal timing and increased risk for externalizing symptoms and aggressive behavior in humans.

## Highlights

1. Adolescence is associated with shifts in agonistic behavioral responses to testosterone.
2. Testosterone increased attacks in prepubertal but not adult males.
3. Testosterone decreased submissive displays in prepubertal but not adult males.
4. Testosterone increased dominance over opponents only in prepubertal males.
5. Testosterone increased flank marking behavior only in adult males.

## 1 Introduction

A dominance hierarchy is established when one animal engages in threatening, chasing, or attacking behaviors, while the opponent responds with defensive or submissive actions (Chase et al., 2002). In social species, dominance confers significant advantages, including prioritized access to resources such as food (Johnston, 1970) and mating opportunities (Huck and Lisk, 1985; Wallen, 1982; Wallen and Goy, 1977). In contrast, in naturally solitary species such as the Syrian hamster (*Mesocricetus auratus*), aggressive interactions are typically driven by territorial defense (Borland and Meisel, 2022; Nowack and Paradiso, 1983). These encounters are resolved when one individual consistently exhibits submissive behavior, thereby yielding to the dominant animal. Unlike the more stable hierarchies observed in social species, dominance relationships among Syrian hamsters are comparatively fluid and subject to change (Borland and Meisel, 2022). Upon initial contact, adult hamsters progress through a series of behaviors that include initial approach, offensive and defensive postures, attacks in attempt to bite, and flank marking. Flank marking occurs when hamsters rub specialized dorsolateral flank glands onto surfaces in their environment (Drickamer et al., 1973; Ferris et al., 1987; Ferris, 1996; Whitsett, 1975). When hamsters are tested in pairs, the individual that exhibits more aggressive displays (e.g., attacks and offensive postures), and fewer submissive displays (e.g., defensive and tail-up postures), is typically considered to be dominant. These dominance relationships are formed quickly and can remain relatively stable across time (De Lorme and Sisk, 2013; Ferris et al., 1987; Grieb et al., 2021; Morrison et al., 2012; Whitten et al., 2023). Across repeated pairings, the dominant male’s overt aggression decreases, whereas flank marking increases, suggesting that flank marking may serve to maintain dominance relationships and reduce the need for continued overt aggression (Ferris et al., 1987). However, given that dominance hierarchies form quickly during a social encounter (Ferris et al., 1987; Goldman and Swanson, 1975; Payne and Swanson, 1970), flank marking may also establish an individual’s dominance status by working in concert with other aggressive displays.

Adolescence is a developmental period characterized by remarkable shifts in both cognitive and social functioning. This stage is often described as a period of social “reorientation,” during which peer relationships gain heightened significance in mammalian species, including humans (De Lorme and Sisk, 2013; Nelson et al., 2016). Social interactions between same-sex conspecifics change dramatically during adolescence. For example, juvenile male Syrian hamsters attack opponents at higher frequencies than adults (Cervantes et al., 2007; Romeo et al., 2003; Wommack et al., 2003), and tend to target attacks toward the head and cheeks of opponents, whereas adult males target attacks toward the lower belly and flank area (Cervantes et al., 2007; Pellis and Pellis, 1988a,1988b; Wommack et al., 2003). In contrast, female Syrian hamsters do not display age-related declines in aggressive behavior and maintain high levels of aggressive behavior throughout adolescence (Taravosh-Lahn and Delville, 2004). Although hamsters are capable of flank marking in response to conspecific odors by postnatal day 22 (Ferris et al., 1996), levels increase dramatically across adolescence during social interactions with same-sex conspecifics (Cervantes et al., 2007; Taravosh-Lahn and Delville, 2004). The current study examines whether adolescent decreases in aggression, and increases in flank marking behavior, are related to changes in responsiveness to steroid hormones before and after pubertal development. Given that aggressive behavior remains stable across the adolescent period in female Syrian hamsters (Taravosh-Lahn and Delville, 2004), we focused our initial investigation in males.

A key biological hallmark of adolescent development is the onset of pubertal secretions of gonadal steroid hormones. The terms puberty and adolescence refer to distinct processes. Puberty involves neuroendocrine maturation that results in the capacity for sexual reproduction. In contrast, adolescence encompasses a broader spectrum of brain and behavioral development. Although distinct processes, the temporal alignment of puberty and adolescence creates the potential for gonadal steroid hormones to not only activate social behaviors in particular contexts, but also shape adolescent brain development via their ability to organize neural networks (Schulz K. et al., 2009; Schulz and Forrester-Fronstin, 2018). In the case of agonistic behavior, prepubertal gonadectomy causes long-lasting alterations in social behavior that are not reversed by testosterone replacement in adulthood. For example, flank marking is typically a testosterone-dependent behavior in adult male Syrian hamsters (Johnston, 1981). However, adult testosterone treatment fails to activate flank marking behavior in males that were gonadectomized prepubertally (De Lorme and Sisk, 2013; Schulz et al., 2006), suggesting that pubertal hormones organize neural networks during adolescence and program flank marking responses to testosterone in adulthood.

Organizational effects of gonadal hormones also mediate pre- to post-pubertal shifts in neuroendocrine and behavioral responses to steroid hormones. Well characterized shifts in steroid negative feedback regulation of the reproductive neuroendocrine axis and steroid facilitation of reproductive behavior occur across puberty and adolescence (Romeo et al., 2002). For example, low doses of testosterone inhibit gonadotropin secretion in juveniles, but not in adults (Richardson et al., 2004; Sisk and Turek, 1983), indicating a pubertal *decrease* in sensitivity to steroid negative feedback regulation of the reproductive axis. In contrast, doses of testosterone that activate reproductive behavior in adults fail to do so in juveniles (Meek et al., 1997; Romeo et al., 2001, 2002; Schulz K. M. et al., 2009), indicating a pubertal *increase* in sensitivity to the activational effects of testosterone on mating behavior. These developmental shifts in sensitivity and responsiveness to testosterone are diminished in prepubertally castrated adult males (Almeida et al., 1987; Schulz et al., 2004), suggesting that pubertal hormones organize neural networks mediating changes in sensitivity across adolescence. Although flank marking behavior, like sexual behavior, is organized by pubertal hormones and testosterone-dependent in adulthood, whether flank marking behavior undergoes a pubertal increase in sensitivity to the activational effects of testosterone is unknown. Likewise, aggressive behavior decreases across adolescence, and adults are relatively insensitive to the behavioral effects of testosterone on aggression (reviewed in Albers et al., 2002; Garrett and Campbell, 1980; Tiefer, 1970), but whether aggression in response to testosterone decreases pre- to post-puberty is not clear.

The current study investigated whether agonistic behavior in response to testosterone changes across puberty and adolescence. Subjects were administered testosterone with the aim of approximating physiological release from the adult testes (Frungieri et al., 1999). As such, behavioral responses reflect the combined androgenic and estrogenic effects of testosterone and its metabolites. Our results indicate that responses to testosterone increase across adolescent development for flank marking behavior, and decrease across adolescence for aggressive and submissive behavioral displays.

## 2 Materials and methods

### 2.1 Experimental design

**FIGURE 1:**
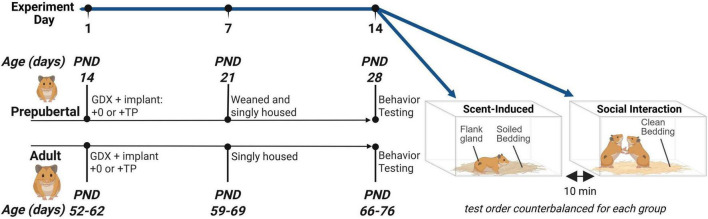
Experimental design. The pubertal rise in gonadal secretions of testosterone begins at approximately 30 days of age and reaches adult levels 3 weeks later around 50 days of age. The timing of gonadectomy and behavioral testing procedures were optimized to occur before or after pubertal development. Subjects were gonadectomized either before puberty at 14 days of age, or after puberty between 52 and 62 days. In the same procedure, subjects were implanted with testosterone propionate (TP) or vehicle pellets, resulting in 4 groups: Prepub + 0, Prepub + TP, Adult + 0, and Adult + TP. Behavioral testing occurred 2 weeks later at 28 days of age (Prepub) or 66–76 days of age (Adult). All subjects were singly housed for 1 week prior to scent-induced flank marking tests and social interaction tests. In the scent-induced flank marking test, subjects were placed into a testing aquarium filled with the soiled bedding of gonad-intact adult males and the number of flank marks were quantified. In the social interaction test, subjects were age- and weight-matched with T-treated male opponents, and the number of aggressive and submissive behavioral displays were quantified. Figure created using Biorender.com.

See Figure 1 for a timeline of experimental procedures. A two-factor between subjects design was employed to assess the effects of Age (prepubertal vs. adult) and Hormone (testosterone vs. vehicle) on agonistic behavior. Endocrine and behavioral puberty occurs between 4 and 7 weeks of age in the male Syrian hamster. Within 3 weeks, testosterone concentrations increase from undetectable to approximately 2–7 ng/mL (Sisk and Turek, 1983; Vomachka and Greenwald, 1979) and adult-typical levels of reproductive behavior are displayed (Cherry, 1987; Miller et al., 1977). To investigate whether agonistic behavioral responses to testosterone change due to developmental processes occurring during puberty and adolescence, the timing of gonadectomy and behavioral testing procedures were optimized to occur before or after pubertal development. Prepubertal subjects were bred in-laboratory and castrated at 14 days of age, and adult subjects were castrated between 52 and 62 days of age, 2 days after their arrival from the vendor. At the time of castration, subjects were implanted with a beeswax pellet containing testosterone propionate (TP) or a control (vehicle) beeswax pellet, resulting in 4 experimental groups: Prepub + 0, Prepub + TP, Adult + 0, and Adult + TP. Two weeks after castration and TP treatment, prepubertal (28 days of age) and adult subjects (66–76 days of age) underwent two behavioral tests separated by a 10-min resting period: 1. A scent test to assess flank marking behavior in response to the soiled bedding of unfamiliar adult males, and 2. A social interaction test in an unfamiliar neutral arena to assess aggressive and submissive behavioral displays during a social interaction with an opponent. Presentation of the scent and social interaction tests was counterbalanced. Opponents were age- and weight-matched within 5 g to a subject and only tested with one subject male. To account for age-dependent differences in circulating testosterone between prepubertal and adult opponents, all opponents were castrated and TP-treated 1 week prior to social interactions. Approximately 1 h after behavioral testing, body weights, blood samples, and flank gland measurements were collected, and animals were euthanized by overdose of sodium pentobarbital.

### 2.2 Animals

All animals were housed in a 14 h light 10 h dark reverse light schedule (lights off at 12:00 EST) to minimize the variation in circadian rhythms and maintain an aggressive reproductive state (Landau, 1975). Food (Teklad Rodent Diet No. 8640, Harlan) and water were available *ad libitum*. Animals were treated in accordance with the NIH Guide for the Care and Use of Laboratory Animals, and all protocols were approved by the Michigan State University Institutional Animal Care and Use Committee.

#### 2.2.1 Prepubertal and adult subjects

Twenty-three prepubertal subjects were bred in laboratory, and 23 adult subjects ranging in age from 50 to 60 days were received from Harlan Sprague-Dawley (Indianapolis, IN) and group housed on arrival. Shipment of adult subjects was timed to occur after pubertal development to minimize the potential effects of adolescent stress on agonistic behavioral responses to gonadal steroid hormones in adulthood (Blaustein et al., 2016; Delville et al., 2003). To avoid potential cohort effects, prepubertal and adult subjects were single housed and behavioral tested in parallel. Prepubertal subjects were weaned and individually housed (37.5 × 33 × 17 cm) at 21 days of age, and adults were individually housed at 59–69 days of age, 7 days prior to behavioral testing.

#### 2.2.2 Prepubertal and adult opponents

Prepubertal opponent males arrived with their dams and littermates from Harlan Sprague-Dawley at 17, 18, and 19 days of age, and were weaned/individually housed following gonadectomy and TP treatment at 21 days of age. Adult opponents were group housed upon arrival at 50–60 days of age and were individually housed following gonadectomy and TP treatment at 59–69 days of age. Like subjects, all opponents were single housed for 7 days prior to social interaction tests.

### 2.3 Surgical procedures

#### 2.3.1 Castration and TP administration

Castrations and testosterone implants were performed in one surgical procedure under isoflurane anesthesia (oxygen with 3–5% isoflurane, flow rate 0.7–1 L/min). Males were administered a subcutaneous injection of the analgesic buprenorphine (0.05 mg/kg) prior to surgery. The testes were pulled through bilateral scrotal incisions, and the testicular veins were tied with suture silk before removal of the testes. The incisions were closed with 9 mm autoclips (Becton Dickinson, 427631). Beeswax pellets containing testosterone propionate (TP) or vehicle were inserted subcutaneously through a 5 mm incision made on the dorsal midline between the scapulae of the animal, and the incision was closed with an autoclip.

#### 2.3.2 Testosterone propionate beeswax pellets

We utilized beeswax as the vehicle for subcutaneous delivery of TP to precisely control the dose administered to subjects and ensure a continuous presence of TP for 14 days (Quispe et al., 2015). Beeswax has been used for administration of hormones in a variety of avian and mammalian species, including Syrian hamsters (Barratt et al., 1977; Bartter et al., 1952; Boersma et al., 2023; Clark et al., 1982; Menendez-Pelaez et al., 1992; Quispe et al., 2015). Testosterone propionate dissolved in 95% ethanol was mixed with melted beeswax (Sigma-Aldrich) at a concentration of 0.05 mg TP/1.0 mg beeswax. The beeswax mixture was allowed to cool and harden in a glass petri dish at room temperature. A metal punch tool was used to extract pellets, and prior to extraction, the beeswax mixture was placed in a −80 freezer for 10 min. The dose of TP administered to animals was determined by the weight of the beeswax pellet, and all pellet weights were verified prior to implantation. Two weeks prior to behavioral testing, subjects were castrated and implanted with a 100 mg beeswax pellet containing 5.0 mg TP or beeswax alone (vehicle). In contrast, 1 week prior to behavioral testing, all opponents were castrated and implanted with a 50 mg pellet containing 2.5 mg TP. TP levels were matched between prepubertal and adult opponents to reduce the likelihood that any behavioral differences between prepubertal and adult subjects were due to variations in the circulating TP levels of the opponents (Evans and Brain, 1974; Payne, 1974; Solomon et al., 2009). Pilot studies determined that beeswax pellets decrease their release of TP over time. Therefore, we adjusted the TP dose administered to opponents to account for their shorter time interval between surgical implants and behavioral testing. This adjustment ensured that during social interactions the testosterone levels of both subjects and their opponents would fall within the range of 2–7 ng/mL that is typically observed in gonad-intact adult male Syrian hamsters (Sisk and Turek, 1983; Vomachka and Greenwald, 1979). Plasma testosterone concentrations were confirmed via radioimmunoassay (Table 1).

**TABLE 1:** Mean plasma testosterone concentrations in prepubertal and adult subjects and their opponents.

Subject treatment group	Subjects	Opponents
	Testosterone (ng/mL)	Testosterone (ng/mL)
Prepub + 0 (*n* = 11)	Undetectable	3.39 (0.51)
Prepub + TP (*n* = 10)	3.98 (0.46)	3.18 (0.37)
Adult + 0 (*n* = 9)	Undetectable	2.73 (0.28)
Adult + TP (*n* = 12)	3.47 (0.37)	2.61 (0.20)

Testosterone levels for all subjects and opponents fell within the typical adult physiological range of 2–7 ng/mL. indicates a marginally significant difference (*p* < 0.1) between TP-treated adult subjects and their opponents. No differences in plasma testosterone levels were found between TP-treated prepubertal and adult subjects. Likewise, no differences were found between the opponents of any subject treatment group.

### 2.4 Behavioral testing and scoring

Behavioral testing began 1 h into the dark phase of the light cycle. Prepubertal and adult subjects were tested sequentially in two counterbalanced conditions: (1) A 10 min social interaction test in which subjects were paired with an age- and weight-matched TP-treated opponent, and (2) a 10 min scent test in which flank marking behavior was observed in response to the soiled bedding of male conspecifics. The social interaction test occurred in a neutral arena unfamiliar to both subjects and opponents. Testing occurred in a neutral arena rather than the subject’s home cage so that defense of a home territory did not bias dominance contests toward subjects. Dyadic encounters in a neutral arena may model interactions between wild hamsters at the outer borders of territories (Borland and Meisel, 2022). For scent tests, soiled bedding was collected from gonad-intact adult males housed 4/cage and stored in an air-tight container. These males were not part of the current study and unfamiliar to all study animals. One cup of soiled bedding was scattered across the floor of the glass aquarium (61 × 32 × 31 cm) immediately before the scent test. For social interaction tests, one cup of clean bedding was scattered across the floor of the aquarium before subjects and opponents were simultaneously placed inside. To ensure that opponents were naïve, they received only one social interaction test. The walls and floor of the aquarium were cleaned thoroughly with 70% ethanol and allowed to dry completely between tests. All tests were video recorded and scored by one experimenter blind to treatment condition. Prior to data collection, intra-rater reliability (90%) was established using a subset of behavioral tests (Table 2).

**TABLE 2:** Behavioral scoring criterion.

Behavior	Scoring criterion
Flank mark	The subject rubs their dorsolateral flank gland against the testing chamber wall.
Close contact	The physical distance between subjects and opponents is within 3 cm.
**Aggressive displays:**	
Attack	The subject moves quickly toward the opponent and attempts to bite.
Paws-on investigation	The subject applies forepaw pressure onto the opponent’s back while actively investigating/sniffing. The opponent stands still during paws-on investigation.
Offensive Posture	The subject stands upright on their hind legs facing the opponent. The opponent must also be displaying an offensive or defensive posture.
**Submissive displays:**	
Tail-up posture	The subject raises their tail (above parallel to the floor) and rounds their back upward (kyphosis). This posture can be assumed when walking or standing still.
Defensive posture	The subject twists their upper body toward the opponent with one or both forepaws outstretched to ward off physical contact.

#### 2.4.1 Social interaction test: subject aggression and win index score

A win index was calculated to investigate whether subjects dominated their opponent during the social interaction. First, an aggression score was calculated separately for subjects and opponents by subtracting an individual’s average number of submissive behavior displays (tail-up + defensive postures/2) from their average number of aggressive behavior displays (attacks + paws-on + offensive postures/3). The subject’s win index was then computed by subtracting the opponent’s aggression score from the subject’s aggression score. Subjects with an index greater than zero were more dominant than their opponent during the social interaction (winner), and subjects with an index less than zero were more submissive than their opponent (loser).

### 2.5 Blood collection and testosterone radioimmunoassay

Approximately 1 h after behavioral testing, subjects were weighed and administered an overdose of sodium pentobarbital (130 mg/kg intraperitoneal). Blood was collected via cardiac puncture into EDTA-coated tubes and centrifuged at°C. Plasma was removed and stored at −20°C until radioimmunoassay. Testosterone concentrations were measured in duplicate 50μL samples within a single assay using the Coat-A-Count Total T Kit (Diagnostic Products, Los Angeles, CA). This assay has been previously validated (Parfitt et al., 1999). The intra-assay CV was 9.2%, and the lower limit of detectability was 0.1 ng/mL.

### 2.6 Flank gland measurement

Flank gland diameter was measured immediately following blood collection. The flank gland (also called the flank organ) is a slightly raised and oval shaped collection of sebaceous scent glands located bilaterally on the dorsolateral flanks. The flank gland increases in diameter and center pigmentation during puberty and is highly responsive to circulating androgen levels in adulthood (Algard et al., 1966; Hamilton and Montagna, 1950). We investigated whether flank gland responsiveness to TP differs between prepubertal and adult subjects. The hair overlying the subject’s right flank gland was shaved before calipers were used to measure the largest diameter of the palpable bulk in millimeters. A central region of dark pigmentation was observed only in TP-treated subjects.

### 2.7 Sample size and experimental attrition

Twenty-three prepubertal and 23 adult subjects underwent behavioral testing procedures, although the following issues resulted in data loss and/or exclusion from analysis. Radioimmunoassay confirmed the failure of a TP implant in one prepubertal subject, and their data were excluded from all analyses. Technical problems with video recording equipment resulted in the loss of two subjects’ social interaction test data, and one subject’s scent-induced test data. Finally, one subject’s social interaction test data were excluded from analysis because they displayed abnormally elevated levels of flank marking behavior. Specifically, this subject’s display of 61 flank marks was 4.97 standard deviations above the mean for all subjects, and more than two times greater than the next highest value of 28 flank marks. Thus, group sizes for analyses ranged between 9 and 12 subjects.

### 2.8 Data analysis

The Kolmogorov-Smirnov test of normality was conducted for each dependent measure. Normally distributed data were analyzed by 2-factor ANOVA or independent *t*-tests. Parametric test results were interpreted using *p*-values, estimates of effect size, and confidence intervals. For ANOVA, effect size was estimated using partial Eta squared values (small effect, η*_*p*_*^2^ = 0.01–0.059; moderate effect, η*_*p*_*^2^ = 0.06–0.139; large effect, η*_*p*_*^2^ = 0.14 and greater), whereas *t*-tests were followed by Cohen’s D estimate of effect size (small effect, *d* = 0.2; moderate effect, *d* = 0.5; large effect, *d* = 0.8). Non-normally distributed data were analyzed by the Kruskal-Wallis or Wilcoxon Signed Rank tests. Mann-Whitney-U tests were used to assess whether the counterbalanced testing order influenced the behavior of subjects during social interactions. Statistical significance was considered *p* < 0.05.

## 3 Results

### 3.1 Testosterone radioimmunoassay

Plasma testosterone concentrations in TP-treated subjects fell within the physiological range of 2–7 ng/mL found in gonad-intact adult male Syrian hamsters (Meek et al., 1997; Sisk and Turek, 1983; Vomachka and Greenwald, 1979). Beeswax TP pellets yielded similar levels of plasma testosterone in prepubertal and adult subjects (Table 1; *Mean Difference* = 0.52 ng/mL, CI 95% [−0.72 to 1.76 ng/mL], *t*(1, 20) = 0.386, *p* = 0.39, *d* = 0.38). Similarly, no differences in plasma testosterone concentrations were found between the opponents that were paired with subjects [Table 1; *F*(3, 38) = 1.10, *p* = 0.36, η*_*p*_*^2^ = 0.08]. We also assessed potential differences in plasma testosterone between subjects and their opponents. For prepubertal males, no significant differences in testosterone concentrations were found between testosterone-treated subjects and their opponents (*Mean Difference* = 0.80 ng/mL, CI 95% [−0.43 to 2.04 ng/mL], *t*(1, 18) = 1.37, *p* = 0.19, *d* = 0.61). For adult males, a marginally significant difference was found between testosterone-treated subjects and their opponents (subjects > partners; *Mean Difference* = 0.86 ng/mL, CI 95% [−0.04 to 1.75 ng/mL], *t*(1, 22) = 2.03, *p* = 0.059, *d* = 0.83). Prepubertal and adult subjects treated with vehicle pellets displayed testosterone concentrations below the lower limit of assay detectability and were not analyzed.

### 3.2 Body weight and flank gland diameter

**FIGURE 2:**
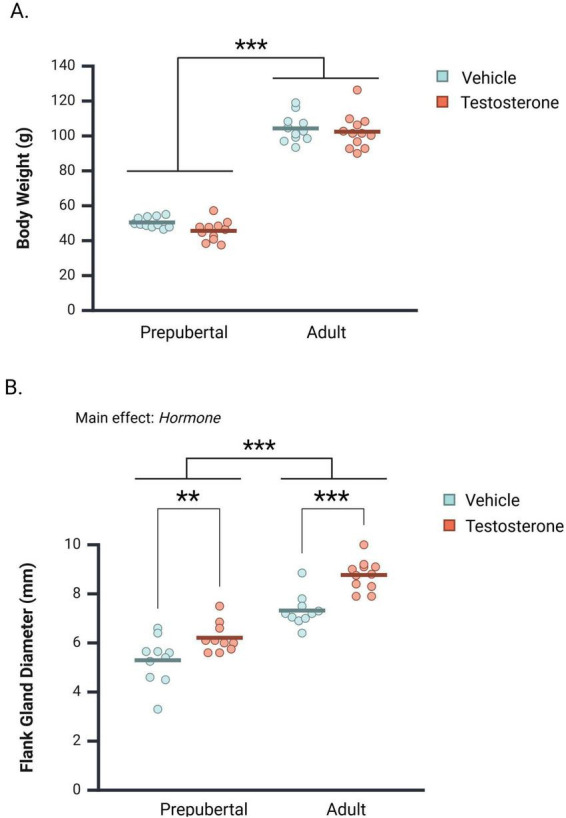
Mean body weight and flank gland diameter in castrated prepubertal and adult subjects. Measurements were taken following behavioral testing. **(A)** Testosterone treatment did not significantly affect body weight in prepubertal or adult subjects. **(B)** Adults exhibited greater flank gland diameters overall, and testosterone administration significantly increased flank gland diameter in both prepubertal and adult subjects. Circles represent individual values, and the bars represent the group mean. **p < 0.01, ***p < 0.001.

As expected, body weights were significantly greater in adults than in prepubertal subjects [Figure 2A; *F(*1, 41) = 671.42, *p* < 0.001, η*_*p*_*^2^ = 0.94]. testosterone treatment did not significantly impact body weight [*F*(1, 41) = 2.47, *p* = 0.124, η*_*p*_*^2^ = 0.06], nor did Age and Hormone interact to influence terminal body weights [*F*(1, 41) = 0.464, *p* = 0.500, η*_*p*_*^2^ = 0.01].

Flank gland diameter was measurable in vehicle- and testosterone-treated subjects, although only testosterone-treated subjects exhibited a darkly pigmented central region. Both Age and Hormone impacted flank gland diameter (Figure 2B). Specifically, testosterone-treatment significantly increased flank gland diameter [*F*(1, 37) = 27.33, *p* < 0.001, η*_*p*_*^2^ = 0.43], and adults exhibited greater flank gland diameters than prepubertal subjects [*F*(1, 37) = 102.78, *p* < 0.001, η*_*p*_*^2^ = 0.74]. Age and Hormone did not interact to influence flank gland diameter [*F*(1, 37) = 1.40, *p* = 0.246, η*_*p*_*^2^ = 0.04]. As such, Hormone significantly increased flank gland diameter in both prepubertal (*Mean Difference* = 0.92 mm, CI 95% [0.26–1.56 mm], *p* < 0.007, η*_*p*_*^2^ = 0.18) and adult subjects (*Mean Difference* = 1.45 mm, CI 95% [0.81–2.10 mm], *p* < 0.001, η*_*p*_*^2^ = 0.36).

### 3.3 Effects of counterbalanced test order

Mann-Whitney U tests were used to assess whether first experiencing the scent or social test influenced subject behavioral displays during social interactions with opponents. Test order did not significantly alter the behavior of subjects during social interaction tests (Table 3). We also conducted Mann-Whitney U tests separately for Prepub + 0, Prepub + T, Adult + 0, and Adult + T groups to confirm that behavior during social interaction tests were not impacted by the order in which testing occurred (no significant differences detected).

**TABLE 3:** Evaluation of counterbalanced testing order results of Mann-Whitney U tests.

Social behavior	Test order	N	Mean	Median	U	Z	*p*-value
Close contact duration (s)	Scent/social	21	396.98	394.86	170.0	−1.270	0.204
	Social/scent	21	335.52	297.84			
Flank marks	Scent/social	21	2.95	0.00	218.5	−0.058	0.954
	Social/scent	21	4.19	0.00			
Attacks	Scent/social	21	5.48	1.00	187.0	−0.888	0.374
	Social/Scent	21	3.29	0.00			
Paws-on displays	Scent/Social	21	6.04	6.00	168.0	−1.582	0.114
	Social/Scent	21	4.20	4.00			
Offensive postures	Scent/Social	21	8.05	6.00	202.0	−0.467	0.640
	Social/Scent	21	9.10	8.00			
Tail-up displays	Scent/Social	21	5.95	1.00	206.5	−0.365	0.715
	Social/Scent	21	7.23	1.00			
Defensive postures	Scent/Social	21	4.81	3.00	166.5	−1.360	0.172
	Social/Scent	21	7.81	4.00			

### 3.4 Flank marking behavior

**FIGURE 3:**
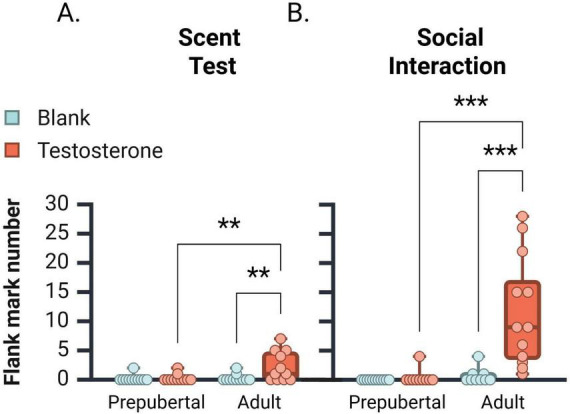
Median number [ ± CI 95%] of flank marks displayed by prepubertal and adult subjects during the scent test **(A)** and social interaction test **(B)**. In both tests, testosterone propionate treatment increased flank marking behavior only in adult subjects. **p < 0.01, ***p < 0.001.

Flank marking data were not normally distributed across groups and required analysis by the Kruskal-Wallis test (Figure 3A). In the scent test, flank marking levels significantly differed between groups [*H*(3, 44) = 11.220, *p* = 0.011], such that testosterone-treated adults displayed significantly more flank marking than vehicle-treated adults (*p* = 0.01), and Prepub + TP (*p* = 0.01) groups. In the social interaction test, a similar pattern of group differences was observed [Figure 3B; *H*(3, 42) = 31.36, *p* < 0.001]. TP-treated adult subjects flank marked more often than Adult + 0 (*p* < 0.001) and Prepub + TP subjects (*p* < 0.001). These data indicate that in either testing context, testosterone activates flank marking behavior in adult but not in prepubertal males.

### 3.5 Close contact with opponent

**FIGURE 4:**
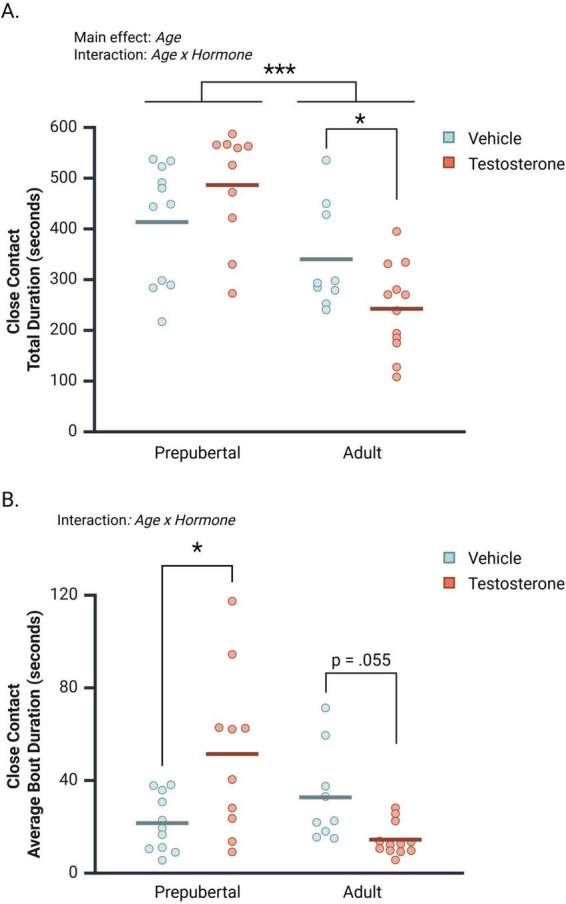
The effects of age and hormone on the duration of close contact between subjects and opponents during the social interaction test. **(A)** Prepubertal subjects spent significantly more time in close contact with opponents than did adults. A significant interaction between Age and Hormone revealed that testosterone propionate (TP) treatment significantly decreased close contact time between adult subjects and opponents, but did not affect prepubertal subject’s time in close contact with opponents. **(B)** Age and Hormone interacted to affect close contact average bout durations. TP-treatment significantly increased close contact bout durations in prepubertal males and decreased close contact bout durations in adult males. Circles represent individual values, and the bars represent the group mean. *p < 0.05, ***p < 0.001.

Prepubertal subjects spent significantly more time in close contact with opponents than did adults [Figure 4A; *F*(1, 38) = 23.70, *p* < 0.001, η*_*p*_*^2^ = 0.38]. An interaction between Age and Hormone also affected total time in close contact [*F*(1, 38) = 6.90, *p* = 0.01, η*_*p*_*^2^ = 0.15]. In prepubertal males, testosterone non-significantly increased close contact duration (*Mean Difference TP* vs. *vehicle* = 73.035 s, CI 95% [−19.81 to 165.88 s], *p* = 0.12). In contrast, testosterone treatment significantly decreased contact duration between adult subjects and their opponents (Figure 4A; *Mean Difference TP vs. vehicle* = −97.650 s, CI 95% [−191.354 to 3.946 s], *p* = 0.04).

The average bout durations of close contact between subjects and their opponents were also examined (Figure 4B). Age and Hormone significantly interacted to influence the duration of close contact bouts [*F*(1, 38) = 13.78, *p* < 0.001, η*_*p*_*^2^ = 0.27]. This interaction was driven by a significant TP-dependent *increase* in contact bout duration in prepubertal subjects (*Mean Difference TP* vs. *vehicle* = 29.81 s, CI 95% [11.38–48.31], *p* = 0.002), and a TP-dependent *decrease* in contact bout duration in adult subjects (*Mean Difference TP* vs. *vehicle* = −18.26 s, CI 95% [−36.893 to 0.38], *p* = 0.055).

### 3.6 Aggressive displays

**FIGURE 5:**
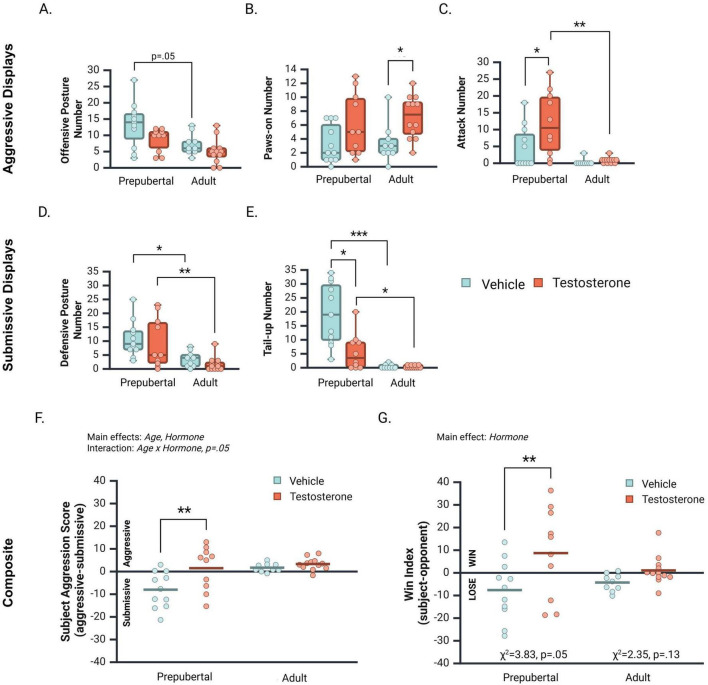
Median number [ ± CI 95%] of aggressive and submissive behaviors displayed by vehicle- and testosterone propionate (TP)-treated prepubertal and adult subjects during a social interaction with an age-matched opponent. **(A)** Vehicle-treated prepubertal subjects displayed the highest number of offensive postures, significantly higher than TP-treated adults. **(B)** TP significantly increased paws-on displays in adult but not prepubertal subjects. **(C)** TP significantly increased attacks in prepubertal but not adult subjects. Prepub + TP males attacked opponents more often than both Adult + 0 and Adult + TP groups. **(D)** TP did not influence defensive postures of prepubertal or adult subjects, however, prepubertal subjects displayed higher levels of defensive postures than adults. **(E)** Tail-up displays were significantly decreased by TP treatment in prepubertal but not in adult subjects. Prepubertal subjects displayed higher levels of tail-up displays than adults. **(F)** The subject’s aggression score reflects the difference between their average aggressive and submissive displays. Aggression scores were influenced by Age, Hormone, and an interaction between Age and Hormone. TP significantly increased aggression scores in prepubertal males, but not in adult males. **(G)** The win index score reflects the difference between aggression scores of subjects and their opponents. TP treatment significantly increased dominance scores overall, and particularly in prepubertal males. TP also increased the proportion of prepubertal subjects that won dominance contests. *p < 0.05, **p < 0.01, ***p < 0.001.

Offensive postures significantly differed between treatment groups [Figure 5A; *H*(3, 42) = 11.03, *p* = 0.01]. Vehicle-treated prepubertal males displayed more offensive postures than vehicle treated adults (*p* = 0.058). Offensive postures did not significantly differ between Prepub + 0 and Prepub + TP (*p* = 0.10), Adult + 0 and Adult + TP (*p* = 0.23), or Prepub + TP and Adult + TP groups (0.124). Treatment group differences were also observed for paws-on displays [Figure 5B; *H*(3, 42) = 9.42, *p* = 0.02]. Testosterone-treated adults displayed significantly more paws-on behavior than vehicle-treated adults (*p* = 0.02). Paws-on displays did not significantly differ between Prepub + 0 and Prepub + TP (*p* = 0.08), Prepub + TP and Adult + TP (*p* = 0.34), or Prepub + 0 and Adult + 0 groups (*p* = 0.82). Attacks on opponents significantly differed between treatment groups [Figure 5C; *H*(3, 42) = 15.60, *p* = 0.001]. Testosterone-treated prepubertal subjects attacked their opponents more often than did vehicle-treated prepubertal subjects (*p* = 0.02), and testosterone-treated adult subjects (*p* < 0.01). No significant difference in attacks was observed between vehicle-treated prepubertal and adult subjects (*p* = 0.08).

### 3.7 Submissive displays

The distribution of defensive postures significantly differed between treatment groups [Figure 5D; *H*(3, 42) = 16.80, *p* < 0.001]. Although no difference was observed between vehicle- and TP-treated prepubertal groups (*p* = 0.25), vehicle-treated prepubertal subjects displayed significantly more defensive postures than vehicle-treated adults (*p* < 0.02). In addition, testosterone-treated prepubertal subjects displayed significantly more defensive postures than testosterone-treated adults (*p* < 0.01; Figure 5D). The distribution of tail-up postures also significantly differed between treatment groups [Figure 5E; *H*(3, 42) = 26.30, *p* < 0.001]. Prepub + 0 subjects displayed significantly more tail-up postures than Prepub + TP (*p* < 0.02) and Adult + 0 subjects (*p* < 0.001). In addition, Prepub + TP subjects displayed significantly more tail-up postures than Adult + TP subjects (*p* < 0.05).

### 3.8 Subject aggression score and win index

Subject aggression scores (average of aggressive displays – average of submissive displays) were significantly higher in adults than in prepubertal subjects (Figure 5F; *F* (1, 38) = 8.40, *p* < 0.01, η*p*^2^ = 0.18). In addition, a main effect of Hormone indicated that testosterone treatment increased subject aggression scores overall [*F*(1, 38) = 7.74, *p* < 0.01, η*_*p*_*^2^ = 0.17]. These main effects were qualified by a marginal interaction between Age and Hormone [*F*(1, 38) = 3.90, *p* < 0.05, η*_*p*_*^2^ = 0.09]. Simple comparisons of the effects of testosterone at each age revealed that testosterone treatment significantly increased aggression scores of prepubertal (Figure 5F; *Mean Difference (T* vs. *vehicle)* = 9.50, CI 95% [3.8–15.20], *p* < 0.01), but not adult subjects (*Mean Difference (T* vs. *vehicle)* = 1.62, CI 95% [−4.12 to 7.35], *p* = 0.57). Notably, most adult subject’s aggression scores were positive, indicating they displayed more aggressive than submissive behaviors during social interactions, irrespective of testosterone treatment.

A subject win index was calculated to investigate whether testosterone treatment increases the likelihood subjects exert dominance over an opponent before and after adolescence (*subject* aggression score - *opponent* aggression score). Two-factor ANOVA revealed that testosterone treatment significantly increased the win index of subjects compared to vehicle treatment [Figure 5G; *F*(1, 38) = 7.92, *p* < 0.01, η*_*p*_*^2^ = 0.17]. This main effect appeared driven by the difference between Prepub + TP and Prepub + 0 subjects (*Mean Difference* = 16.34, CI 95% [5.36–27.32], *p* < 0.01), more so than by the difference between Adult + TP and Adult + 0 subjects (*Mean Difference* = 5.35, CI 95% [−5.73–16.43], *p* = 0.34). Age did not significantly impact the win index [Figure 5G; *F*(1, 38) = 0.31, *p* = 0.58, η*_*p*_*^2^ = 0.01], nor did Age and Hormone significantly interact [*F*(1, 38) = 2.03, *p* = 0.16, η*_*p*_*^2^ = 0.05]. We also tested whether hormone status influenced the proportion of prepubertal and adult subjects who won dominance over opponents. In prepubertal males, testosterone-treated subjects were significantly more likely to win dominance over an opponent than vehicle-treated subjects (χ^2^ (1, 21) = 3.83, *p* = 0.05). In contrast, testosterone treatment did not significantly alter the proportion of adult males who won dominance over opponents (χ^2^ (1, 21) = 3.83, *p* = 0.125). Taken together, these data suggest that testosterone increased the competitive motivation to dominate an opponent to a greater extent in prepubertal than adult males.

### 3.9 Opponent attacks and tail-up postures

**FIGURE 6:**
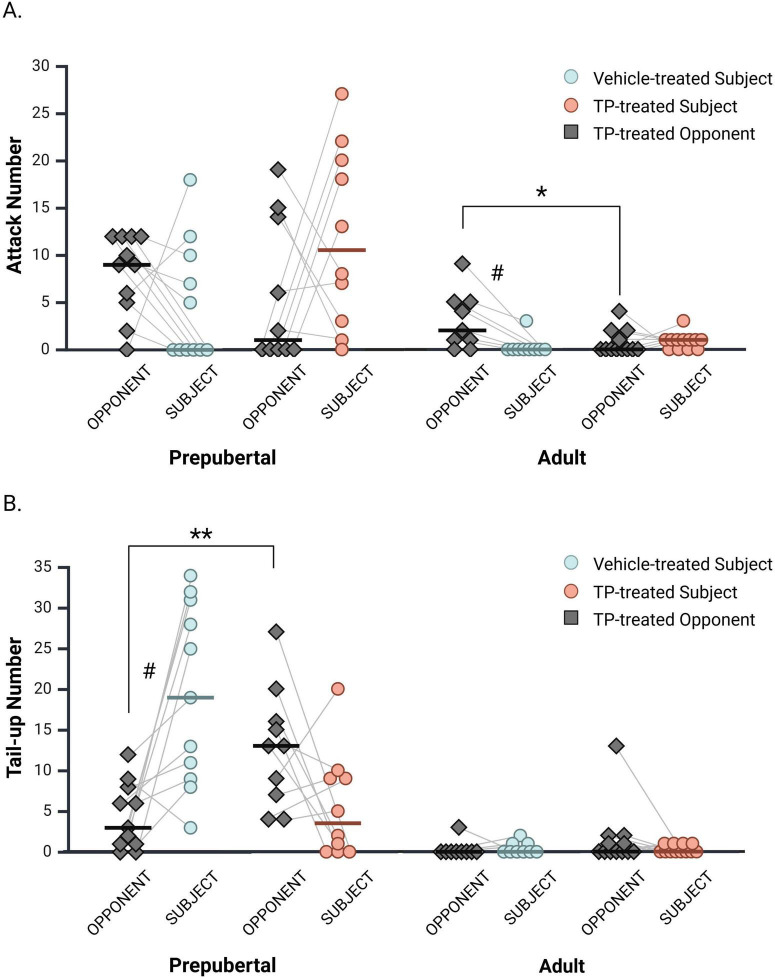
Opponent attacks and tail up displays during interactions with vehicle- and TP-treated subjects. **(A)** Adult opponents attacked vehicle-treated subjects more than TP-treated subjects. A significant difference in attacks was also observed between vehicle-treated adult subjects and their opponents, but not between TP-treated adult subjects and opponents. Prepubertal opponent attacks did not differ depending on subject hormone status, nor did attack frequencies differ within subject and opponent dyads. **(B)** Prepubertal opponents displayed more tail-up postures during interactions with TP-treated subjects than with vehicle-treated subjects. In prepubertal dyads, vehicle-treated subjects displayed significantly more tail-up postures than did their opponents, whereas tail-up displays did not differ between TP-treated prepubertal subjects and their opponents. In adults, opponent tail-up postures did not change depending on subject hormone status, nor were differences observed within subject and opponent dyads. *p < 0.05, **p < 0.01. # denotes a significant difference within subject and opponent dyads.

Previous studies demonstrate that resident male hamsters attack gonad-intact intruders at higher levels than castrated intruders (Evans and Brain, 1974; Payne, 1974; Solomon et al., 2009), suggesting that an opponent’s hormone status influences resident aggression. Given this, all opponents in the current study were gonadectomized and TP-treated to prevent opponent hormone status from confounding the interpretation of behavioral differences between prepubertal and adult subjects. Nevertheless, differences in opponent attacks on vehicle- and TP-treated subjects may also influence behavioral differences observed between subject groups. Therefore, we examined the effects of subject hormone status on opponent attacks and tail-up postures (Figure 6). A comparison of opponents matched with vehicle or TP-treated subjects revealed that adult opponents attacked vehicle-treated subjects more often than TP-treated subjects [Figure 6A; *H*(1, 21) = 4.63, *p* = 0.031]. In contrast, prepubertal opponents did not differ in their attacks of vehicle- and TP-treated subjects [*H*(1, 21) = 1.10, *p* = 0.30]. We also evaluated attacks within subject and opponent dyads separately for each experimental group using Wilcoxon Signed Rank Tests for paired samples. Adult opponents displayed more attacks (median = 2; IQR = 0.5–5.0) than Adult + 0 subjects (median = 0, IQR = 0–0; *z* = −2.38, *p* = 0.018). No differences between subject and opponent attacks were observed within Adult + TP (*p* = 0.66), Prepub + 0 (*p* = 0.15), or Prepub + TP (*p* = 0.15) groups.

For tail-up displays, prepubertal opponents matched with TP-treated subjects displayed more tail up postures than prepubertal opponents matched with vehicle-treated subjects [Figure 6B; *H*(1, 21) = 8.20, *p* < 0.01]. Adult opponent tail up displays did not differ when paired with vehicle- or TP-treated subjects [Figure 6B; *H*(1, 21) = 1.80, *p* = 0.18]. Within subject and opponent dyads, Prepub + 0 subjects displayed more tail-up postures (median = 19; IQR = 9.0–31.0) than their opponents (Figure 6B; median = 3; IQR = 1.0−8.0; *z* = −2.70, *p* < 0.01). No differences between subject and opponent tail-up postures were observed within Prepub + TP (*p* = 0.11), Adult + 0 (*p* = 0.71), or Adult + TP (*p* = 0.30) pairings.

## 4 Discussion

The current study compared the behaviors of prepubertal and adult males to test the hypothesis that a shift in agonistic responses to testosterone occurs following puberty in male Syrian hamsters. Given that aggressive behavior declines during adolescence and is not dependent on testosterone in adulthood, we hypothesized that responsiveness to testosterone would decrease following pubertal maturation. Supporting this hypothesis, testosterone treatment increased attack behavior in prepubertal but not adult males. Interestingly, submissive behaviors also declined across adolescence, and testosterone treatment reduced submissive tail-up displays only in prepubertal males. Thus, both aggressive and submissive behavioral displays showed reduced responsiveness to testosterone following pubertal development. Given that flank marking behavior increases across adolescence and is testosterone dependent in adult males, we hypothesized that flank marking in response to testosterone would increase following pubertal development. During both social interaction and scent tests, testosterone facilitated flank marking behavior *only* in adult subjects, indicating that behavioral responsiveness to testosterone requires developmental processes occurring during puberty and adolescence. Collectively, these results provide the first evidence that changes in responsiveness to steroid hormones occur across puberty and adolescence for male agonistic behaviors, much like the well-established shifts in neuroendocrine and reproductive behavioral responses to steroid hormones that occur pre- to post-pubertally.

Adolescent changes in agonistic behavior and responses to testosterone were evaluated in the context of interactions with age-matched opponents in a neutral test arena. To investigate whether testosterone treatment facilitated dominance over an opponent, we calculated a win index by subtracting the opponent’s aggression score (mean aggressive displays – mean submissive displays) from each subject’s aggression score. Testosterone treatment significantly increased win index values in prepubertal but not adult subjects. Thus, steroid-dependent alterations in agonistic behavior facilitated dominance wins in prepubertal but not adult subjects. The differential effects of testosterone on prepubertal and adult dominance scores may also reflect adolescent changes in social motivation and sensitivity to environmental context. For example, adult male rats interact at higher levels in familiar environments than in unfamiliar environments, whereas in prepubertal males, social interactions do not change depending on the familiarity of the environment (Primus and Kellogg, 1989). In addition, prepubertal gonadectomy prevents the development of the adult pattern of social interaction in familiar vs. unfamiliar environments (Primus and Kellogg, 1989, 1990). In the current study, testing occurred in an unfamiliar neutral arena rather than the subject’s home cage so that defense of a home territory did not bias dominance contests toward subjects. In line with previous studies in rats, prepubertal subjects spent significantly more time in close contact with opponents than did adults. Furthermore, testosterone treatment increased social contact bout durations in prepubertal males, and decreased contact durations in adults. Thus, in addition to the overall decline in social motivation observed across adolescence, testosterone treatment also promoted unique social strategies in an unfamiliar environment before and after puberty. In adulthood, testosterone promoted a strategy characterized by decreased contact with opponents and increased scent communication via flank marking behavior, whereas prior to puberty, testosterone promoted a social strategy characterized by increased physical interaction aimed at dominating an opponent.

Flank marking is a testosterone-dependent form of scent communication utilized by adult male Syrian hamsters to communicate dominance status (Ferris et al., 1987; Johnston, 1981). Since the effects of testosterone on behavior are often context-dependent, we evaluated flank marking behavior in two different contexts: during social interactions and in response to male odors alone. In both contexts, testosterone treatment stimulated flank marking behavior in adult males, but not in prepubertal males. This suggests that testosterone’s effects on flank marking behavior do not appear until after puberty and adolescence. We also evaluated whether peripheral flank glands were responsive to the presence of testosterone prior to puberty and found that testosterone treatment increased flank gland diameters in both prepubertal and adult males. Therefore, the lack of behavioral response to testosterone in prepubertal males is not likely due to insensitivity of peripheral flank gland tissues. Instead, the inability of testosterone to activate flank marking behavior before adolescence is likely due to unresponsive behavioral neural circuits at this age, which is also the case for sexual behavior in this species (Schulz K. M. et al., 2009). Testosterone’s unique effects on flank marking and aggression may also indicate these behaviors have distinctive underlying neural circuits within the social behavior network that are differentially regulated by testosterone before and after puberty.

Previous studies using the resident-intruder paradigm demonstrate that a conspecific’s hormonal status (e.g., gonad-intact, or castrated) can influence the resident’s aggressive behavior (Evans and Brain, 1974; Payne, 1974; Solomon et al., 2009). To avoid confounding our interpretation of behavioral differences between prepubertal and adult subjects, we controlled testosterone levels in opponents. However, given the reciprocal nature of dominance interactions, we also examined whether opponents displayed differential aggression toward vehicle- or TP-treated subjects. In prepubertal males, opponent attack frequencies did not differ between treatment groups. Nonetheless, prepubertal opponents exhibited more submissive tail-up postures during interactions with TP-treated subjects, indicating a possible sensitivity to TP-induced behavioral cues. In general, these findings suggest that differences observed between vehicle- and TP-treated prepubertal subjects were not driven by variations in attacks received from opponents. In adult males, however, opponents directed more attacks toward vehicle-treated subjects than toward TP-treated subjects. This pattern contrasts with previous resident-intruder test findings, where resident aggression was more prominent in the presence of intact or hormone-treated opponents (Evans and Brain, 1974; Payne, 1974; Solomon et al., 2009). The increased aggression directed at vehicle-treated adults raises the possibility that some behavioral differences between adult treatment groups may be partially explained by differential levels of attacks received from opponents. Specifically, increased attacks on vehicle-treated subjects may have suppressed certain aggressive behaviors, such as paws-on displays. To further investigate the role of hormone-dependent reciprocity in agonistic interactions, our future studies will systematically manipulate the hormone levels of both individuals within a dyad. This approach will allow for a controlled analysis of dyad composition (e.g., hormone–hormone, hormone–vehicle, vehicle–vehicle) and its influence on aggressive and submissive behaviors.

Previous studies investigating age-related changes in aggressive behavior demonstrate that adolescence is associated with a decrease in attack frequency and a change in the bodily targets of attack (Cervantes et al., 2007; Pellis and Pellis, 1988a,1988b; Wommack et al., 2003). These previous studies utilized gonad-intact males and assessed aggression in both neutral and home territories across time. Our findings using a cross-sectional design (before and after adolescence) and testing subjects in a neutral arena support previous reports of an age-related decline in attack frequency. However, to our knowledge, we are the first to report that testosterone *facilitates* attacks in prepubertal males. Although this finding conflicts with a previous report that a 1-wk testosterone treatment fails to influence prepubertal attacks on intruders in their home cage (Romeo et al., 2003), differences in both the duration of testosterone treatment (1 vs. 2 weeks) and the testing environment (home territory vs. neutral arena) likely explain the differential effects of testosterone on attacks between these studies. We also report here that testosterone treatment decreased tail-up submissive postures in prepubertal males. In contrast, adults displayed very few tail-up or defensive postures, irrespective of testosterone treatment. The overall higher levels of submissive behavior displayed by prepubertal males suggests that submissive behavioral displays decrease during adolescent development, much like the well-documented decrease in attacks across the pre- to post-adolescent period (reviewed in Delville et al., 2003).

The neural mechanisms underlying prepubertal and adult differences in agonistic behavioral responses to testosterone are currently unknown. Although prepubertal males were more responsive than adults to the effects of testosterone on attacks and tail-up displays, it is unclear whether the lack of responsiveness in adult subjects reflects an adolescent decrease in neural responsiveness to testosterone during adolescent development. Interestingly, gonadectomized and testosterone-treated prepubertal males display higher densities of androgen receptor (AR) than adults within regions of the social behavior network that regulate reproductive and aggressive behavior (Meek et al., 1997). Specifically, AR densities are higher in prepubertal than adult males within the medial amygdala (MeA), medial preoptic nucleus (MPNmag), and bed nucleus of the stria terminalis (BNST). Thus, it is possible that adolescent decreases in androgen receptor within this network contributes to the differences in behavioral responsiveness to testosterone observed between prepubertal and adult males. Given that the effects of testosterone on aggression are mediated by social context in adulthood (Oliveira, 2009; Solomon et al., 2009; Whitten et al., 2024), it is also likely that differences observed between prepubertal and adult males relate to adolescent development of corticolimbic circuits underlying social cognition (Blakemore, 2008; Murray et al., 2024) and threat perception (Spielberg et al., 2015). Adolescent changes in corticolimbic circuits promote the context-appropriate expression of social behavior (De Lorme et al., 2013; De Lorme and Sisk, 2013), and may also change the context-dependent effects of testosterone on aggressive and submissive behavioral displays in adulthood.

A complex relationship exists between the timing of puberty and the timing of brain sensitivity to the organizing actions of gonadal steroid hormones, and scientific tests of these relationships are sorely needed. Schulz K. M. et al. (2009) proposed a model of decreasing brain sensitivity to the organizing actions of gonadal steroid hormones across adolescent development. This model originated from studies of male hamster reproductive behavior demonstrating that the window of sensitivity to testosterone’s organizing effects closes after adolescence, and that testosterone treatments before puberty have a greater impact on adult reproductive function than treatments during the typical pubertal period (Schulz K. M. et al., 2009). Although a direct test of this model has not been conducted for male agonistic behavior, it is important to consider whether the behavioral outcomes in the current study reflect both organizational and activational effects of prepubertal testosterone treatment. The pubertal rise in gonadal hormones typically spans a 21 day time period (Sisk and Turek, 1983; Vomachka and Greenwald, 1979), therefore, the 14-d prepubertal testosterone treatment may have been sufficient to induce organizational effects on behavior. Additional studies are needed to determine whether prepubertal testosterone treatment exerts long-term effects on aggressive behavior.

## 5 Conclusion

We provide here the first evidence that shifts in agonistic behavioral responses to testosterone occur across puberty and adolescence in male rodents. Testosterone treatment increased attack behavior in prepubertal but not adult males. In adults, testosterone stimulated high levels of flank-marking behavior, but it did not induce flank marking in prepubertal males. Several studies in humans have linked early pubertal timing with increased adolescent aggression and externalizing symptoms (Chen and Raine, 2018; Cota-Robles et al., 2002; Ge et al., 2006; Glowacz and Bourguignon, 2015; Najman et al., 2009). Our findings support the possibility that early exposure to pubertal hormones increases aggressive behavior and risk for externalizing symptoms during adolescence.

## Data Availability

The raw data supporting the conclusions of this article will be made available by the authors, without undue reservation.
